# Use of confocal microscopy imaging for in vitro assessment of adipose‐derived mesenchymal stromal cells seeding on acellular dermal matrices: 3D reconstruction based on collagen autofluorescence

**DOI:** 10.1111/srt.13103

**Published:** 2021-09-23

**Authors:** Alessia Paganelli, Elisabetta Tarentini, Luisa Benassi, Daniel Scelfo, Alessandra Pisciotta, Elena Rossi, Cristina Magnoni

**Affiliations:** ^1^ Surgical, Medical and Dental Department of Morphological Sciences related to Transplant, Oncology and Regenerative Medicine Division of Dermatology University of Modena and Reggio Emilia Modena and Reggio Emilia Italy; ^2^ PhD Program in Clinical and Experimental Medicine University of Modena and Reggio Emilia Modena and Reggio Emilia Italy

**Keywords:** acellular dermal matrix, ADSC, autofluorescence, confocal microscopy, MSC, wound healing

## Abstract

**Background:**

Both mesenchymal stromal cells (MSCs) and acellular dermal matrices (ADMs) represent fascinating therapeutic tools in the wound healing scenario. Strategies aimed at combining these two treatment modalities are currently under investigation. Moreover, scarcity of quantitative, nondestructive techniques for quality assessment of engineered tissues poses great limitations in regenerative medicine and collagen autofluorescence‐based imaging techniques are acquiring great importance in this setting.

**Objective:**

Our goals were to assess the in vitro interactions between ADSCs and ADMs and to analyze extracellular‐matrix production.

**Methods:**

Adipose‐derived MSCs (ADSC) were plated on 8‐mm punch biopsies of a commercially available ADM (Integra®). Conventional histology with hematoxylin‐eosin staining, environmental scanning electron microscopy, and confocal‐laser scanning microscopy were used to obtain imaging of ADSC‐seeded ADMs. Collagen production by ADSCs was quantified by mean fluorescence intensity (MFI), expressed in terms of positive pixels/field, obtained through ImageJ software processing of three‐dimensional projections from confocal scanning images. Control conditions included: fibroblast‐seeded ADM, ADSC‐ and fibroblast‐induced scaffolds, and Integra® alone.

**Results:**

ADSCs were efficiently seeded on Integra® and were perfectly incorporated in the pores of the scaffold. Collagen production was revealed to be significantly higher when ADSCs were seeded on ADM rather than in all other control conditions. Collagen autofluorescence was efficiently used as a surrogate marker of ECM production.

**Conclusions:**

Combined therapies based on MSCs and collagenic ADMs are promising therapeutic options for chronic wounds. Not only ADSCs can be efficiently seeded on ADMs, but ADMs also seem to potentiate their regenerative properties, as highlightable from fluorescence confocal imaging.

## 1 INTRODUCTION

Wound healing is an evolutionary conserved process composed of four sequential yet overlapping phases: hemostasis, inflammation, proliferation, and remodeling.^1^ This peculiar sequence of events is strictly regulated and driven by skin resident keratinocytes, fibroblasts, inflammatory cells, and soluble mediators, such as cytokines and growth factors.^2^ Despite the recent advances achieved in this field, skin can only reach 70% of its original strength in a normal wound healing process and invariably heals with scarring.^3^ Different techniques have been introduced in the last decades both to minimize scar formation and to reduce healing time. However, the use of advanced wound‐care strategies is still unsatisfactory for the long healing time, the frequent recurrence of ulcers (60–70%), the poor quality of the scar tissue, and the high costs for patient hospitalization.^4^

There is a variety of different conditions characterized by full‐thickness skin wounds (burns, demolitive surgery, vascular, and/or diabetic ulcers) in which the dermal and epidermal compartments are both totally disrupted. In those cases, conventional surgery with split‐thickness skin graft was often used as first‐line treatment for coverage of the damaged area in the past.^5^ Despite the proven efficacy of this strategy, lack of dermal component inevitably determines the formation of contractures and adhesions during the wound healing process, determining scar formation. In fact, dermal elements are crucial for their role in granulation tissue formation, remodeling, and reepithelization.

New approaches to chronic wounds and ulcers have been proposed in the last decades, including the use of full‐thickness skin grafts and bioengineered dermal scaffolds.^6^, ^7^ In theory, the ideal scaffold should be a biologically active matrix capable of preventing spontaneous scar formation and inducing regeneration in injured tissues.^8^, ^9^ With regard to this, acellular dermal matrices (ADMs) have been proposed as a promising tool in the field of regenerative medicine and wound healing.^6^, ^10^

Scaffold materials may vary from temporary interfaces to permanently incorporated dermal elements and can be either synthetic or natural.^6^, ^11^ Synthetic materials certainly represent a fascinating tool for their predictable mechanical and physical properties, but sometimes can elicit a foreign body like reaction. Therefore, natural scaffolds composed of hyaluronic acid and purified collagen have been investigated as alternatives to synthetic scaffolds. In particular, collagen‐based ADMs are today widely used in the dermatological setting.^11^, ^12^

Currently, cellular strategies are also under investigation for the treatment of cutaneous wounds. Several works already reported on the possibility of using fibroblast‐derived extracellular matrix (ECM) scaffolds for wound healing in skin injuries.^13^, ^14^ However, terminally differentiated fibroblasts lack regenerative capacity. On the contrary, mesenchymal stromal cells (MSCs) are a subset of multipotent cells present in tissues of mesenchymal origin that can acquire a fibroblast‐like phenotype.^15^, ^16^ MSCs are currently considered important candidates in tissue engineering because of their plasticity. MSCs include many different cell types, probably the most widely studied being the bone‐marrow stromal stem cells (BMSCs). MSC sources also include adipose tissue, placenta, umbilical cord, skeletal muscle, synovium, dental tissue, and many others.

Adipose‐derived stromal cells (ADSCs), in particular, hold several advantages over BMSCs as a therapeutic cell source. First of all, safety of the isolation procedure.^17^, ^18^ Second, the average frequency of MSCs in processed lipoaspirate is much higher than in BM (approximately 2% vs. 1 in 25 000–100 000 nucleated cells). Those characteristics bring the possibility of transplanting freshly isolated ADSCs without the need of cell culturing; therefore not only lowering the risk of microbial contamination, but also preventing cellular alterations and processing errors.

ADSC‐derived scaffolds seem to have ideal mechanical and biological properties. In fact, being capable of differentiating toward a fibroblast‐like phenotype, ADSCs can produce all ECM components, potentially completely restoring dermal architecture.^19^ Our group already isolated and characterized human ADSCs obtained from lipoaspirates and evaluated their ability to secrete crucial components of the ECM upon stimulation.^20^ ADSCs have also been proven to stimulate wound repair and to accelerate healing. However, previous works showed that isolated cells are hardly able to organize themselves spontaneously to form complex tissue structures in the absence of a three‐dimensional matrix that guides and stimulates the entire process.^21^ In fact, tissue regeneration requires a support (scaffold) that emulates the ECM and provides a temporary guide for cell migration and proliferation.^22^ Organotypic models of wound healing demonstrated that integrin αV is expressed very early, mimicking the early phases of wound healing.^19^ In fact, integrins belonging to αV and β1 families promote cell motility through linking to fibronectin, collagen, vitronectin, or laminin and other molecules present in the ECM. In the same in vitro experimental models, epithelization of the dermal matrix occurred in parallel with collagen IV deposition at the dermal‐epidermal junction, thus attesting initial basal membrane formation. Wound‐healing assays also suggest that ADSCs produce some soluble factors responsible for their effect on keratinocytes.^23^, ^24^

New studies aimed at combining the use of MSCs and other biomaterials in tissue repair and regeneration are today ongoing.^22^ ADMs seem to provide an optimal scaffolding material for MSCs, especially if considering their promoting role in angiogenesis and revascularization. Given the importance of those strategies in the field of wound healing and regenerative medicine, the aim of our work was to assess the in vitro interactions between ADSCs and ADMs. In particular, the main goal of the present study was to clarify whether ADSCs could integrate and proliferate when seeded in ADMs. Moreover, we also aimed at assessing if ADMs potentiate ADSC‐mediated collagen production both from a qualitative and a quantitative point of view.

## 2 MATERIALS AND METHODS

### 2.1 Cell isolation, characterization, and culturing

In accordance with previously published data, we isolated and characterized ADSCs and fibroblasts using the protocol already validated by our group and approved by the institutional review board (University of Modena and Reggio Emilia, Protocol Number CE2690/16).^20^ The investigation was conducted in accordance with the Declaration of Helsinki and all the subjects provided written informed consent prior to any surgical procedure and further cell manipulation, according to the rules of good clinical practice. Briefly, tissue obtained from different donors was digested with Collagenase type I 0.75 mg/ml (Sigma‐Aldrich, St. Louis, MO, USA) in HBSS supplemented with Hepes 20 mM and 1.5% BSA and subsequently underwent centrifugation at 1500 rpm for 5 min. The cell pellet (identified as the SVF, *stromal vascular fraction*) was then resuspended in erythrocyte lysis buffer and plated into 75 cm^2^ flasks in culture medium (DMEM/Ham's F12, 20% FBS, 1% PSA, and 4 mM glutamine) enriched with 10 ng/ml basic fibroblast growth factor (bFGF) (Sigma‐Aldrich, St. Louis, MO, USA), defined as stromal medium. After three to five passages in culture, ADSCs were characterized by flow cytometry (Epics cytofluorimeter, Beckman Coulter), using the following mAbs: anti‐CD73 (Abcam, Cambridge, UK), anti‐CD90‐FITC (BD Pharmingen, San Diego, CA, USA), anti‐CD105‐FITC (RD Systems, Minneapolis, MN, USA), and anti‐CD45‐FITC (BD Pharmingen, San Diego, CA, USA).

### 2.2 ADMs

Integra® (LifeSciences, Plainsboro, New Jersey) is a commercial porous matrix of cross‐linked bovine tendon collagen fibers and glycosaminoglycan (chondroitin‐6‐sulfate) from shark cartilage. This acellular dermal substitute enables the regeneration of full‐thickness wounds and is commonly used as a first step in two‐step surgical procedures. The ADM is applied directly onto the wound after surgical resection or debridement, and followed by a split‐thickness skin graft, generally performed 3 weeks later. Integra® is a two‐layer device: while the collagenic component directly contacts the wound bed, a silicone sheet forms an outer layer, and has a barrier function during the first weeks after grafting.

### 2.3 Cell plating

Cell‐seeded dermal scaffolds were assembled introducing ADSCs or fibroblasts into the previously described ADM. Dermal substitutes were rinsed with 0.9% NaCl and subsequently washed in Hank's balanced salt solution for 1 hour at 37°C to remove the residual preservatives (e.g. glutaraldehyde). Eight‐millimeter punch biopsies of Integra® were transferred in 96‐multiwell plates and incubated with culture medium. Then, ADSCs and fibroblasts were seeded on ADM punches at a density of 5 × 10^5^ cells/well. Afterward, cultures were submerged with stromal medium and cells were maintained in culture for 3 weeks, with medium being replaced every 2–3 days. Fibroblasts and ADSCs alone were also cultured for 28 days in the presence of 250 μM ascorbic acid, as already described by our group. Those fibroblast‐ and ADSC‐derived scaffolds were used as a control.

### 2.4 Standard imaging of ADSC integration in ADMs

For histochemical analysis, the ADSC‐seeded ADMs were fixed in 4% buffered paraformaldehyde pH 7.4 overnight. After dehydration, scaffolds specimens were embedded in paraffin, and 4‐μm sections were cut, deparaffinized, rehydrated, and stained with HE for conventional morphological analysis. Axioskope 40 microscope (Zeiss, Germany) and Nikon digital camera (Nikon Corp., Tokyo, Japan) were used for image acquisition.

ADSC‐seeded ADMs were also investigated through environmental scanning electron microscopy (ESEM). After washing in Phosphate‐buffered saline (PBS), constructs were fixed with 2.5% glutaraldehyde at 0°C, dehydrated, and dried in a critical‐point dryer. Afterward, they were observed under a Quanta200 ESEM (FEI, Hillsboro, OR, USA).

### 2.5 3D reconstruction of cell‐seeded constructs

The entire 8‐mm punch was incubated with falloidin‐TRITC (Abcam, UK) previously diluted 1:1000 in Dimethyl sulfoxide (DMSO) and counterstained with 1 μg/ml 4′,6‐ diamidino‐2‐phenylindole (DAPI, Sigma‐Aldrich). Then, slides were mounted with FluoroMount anti‐fading medium (Sigma‐Aldrich) before evaluation under a Nikon A1 confocal laser scanning microscope.

**FIGURE 1:**
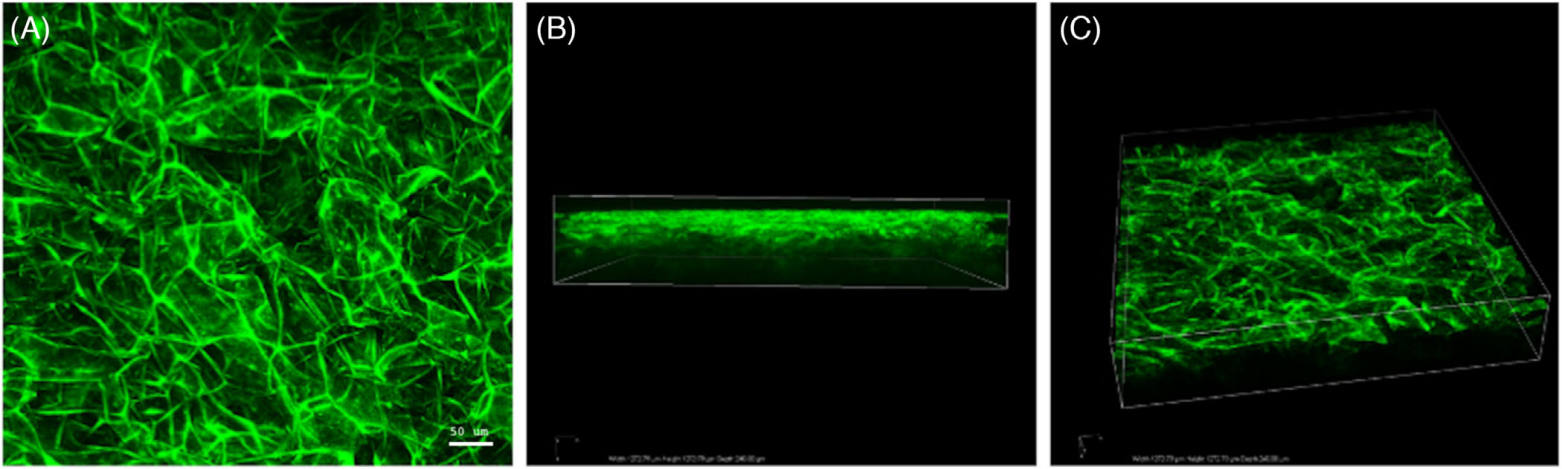
Confocal‐imaging‐based 3D reconstruction of a full‐thickness sheet of Integra® alone. Green color is not given by a specific staining but comes from collagen autofluorescence. Magnification 20x

For collagen evaluation, no specific immunofluorescence stain was performed. Autofluorescence of collagen fibers was detected by exciting the samples with a 488 nm argon gas laser, with a green color emission (see Figure 1). The confocal serial sections were processed with ImageJ software to obtain three‐dimensional projections, and image rendering was performed using Adobe Photoshop Software. Collagen density was quantified by mean fluorescence intensity (MFI), expressed in terms of positive pixels/field, obtained from measurements in three different areas (13 000 μm^2^) of three randomly selected sections from every tissue sample.

### 2.6 Statistical analysis

Statistical analysis was done using STATA program version 14 (StataCorp LP 4905 Lakeway Drive College Station, TX, USA). Numerical data were expressed as mean and standard deviation. Student's *t*‐test was used to assess differences between two conditions. *p*‐Values ≤ 0.05 were considered statistically significant

## 3 RESULTS

ADSCs were successfully generated from adipose tissue contained in discarded material obtained during dermatologic surgical procedures. As already described in previous works, ADSCs were defined as CD45‐negative, CD90, CD73, and CD105 positive cells (data not shown).^19^, ^20^

**FIGURE 2:**
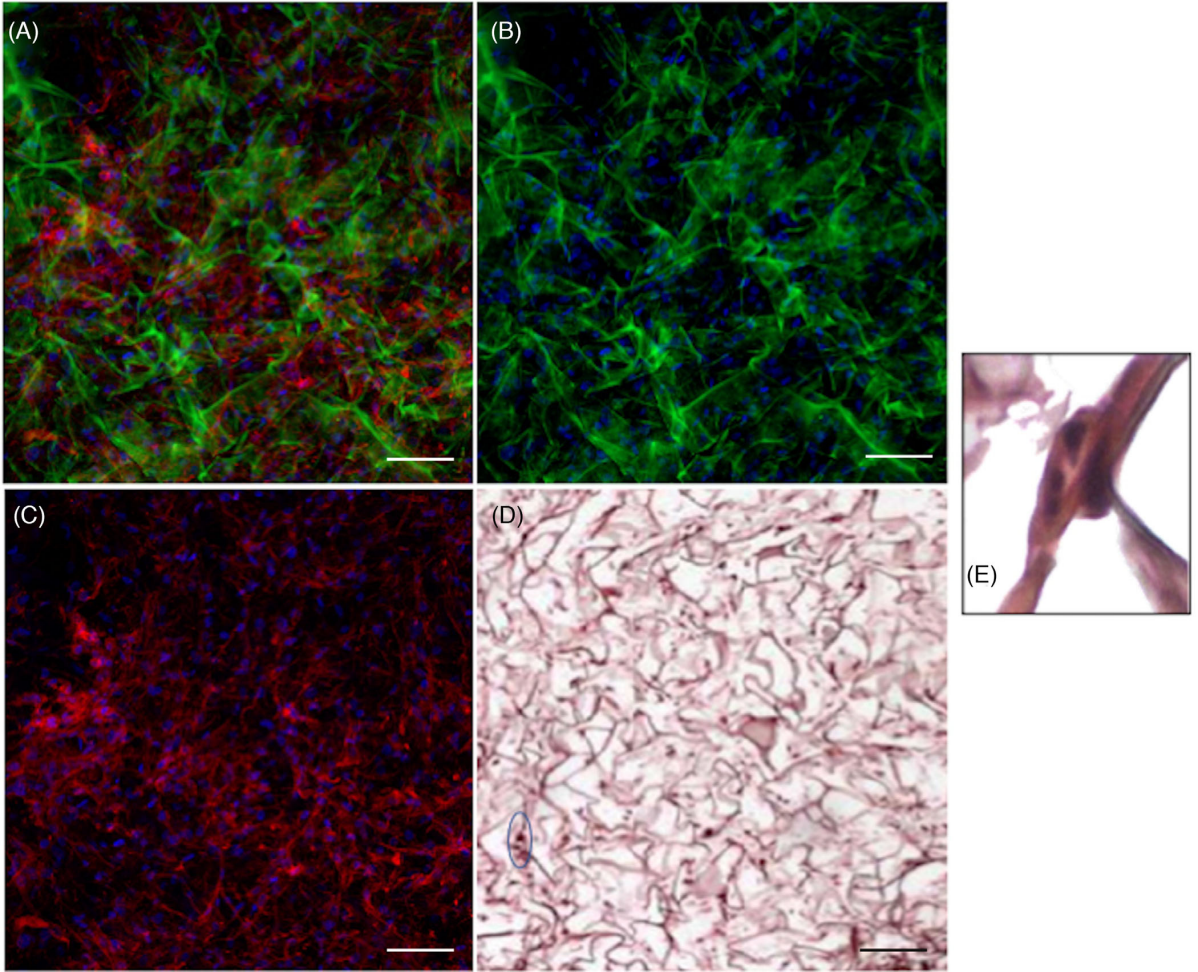
ADSC‐seeded dermal scaffold. Cells are visibly incorporated in the pores of the ADM. (A)–(C) Samples observed by a Nikon‐A1 confocal laser scanning microscope. Green: collagen; red: phalloidin; blue: DAPI. (D) Conventional HE stain. (E) Enlarged detail of ADSC‐seeded scaffold at 40× magnification. The blue square identifies cells in mitosis. Magnification 20x. Scale bar 50 μm

**FIGURE 3:**
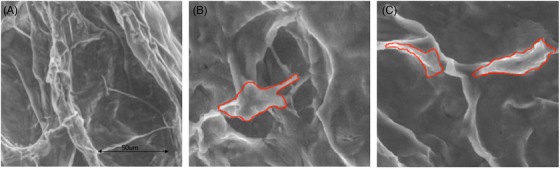
ESEM images of ADM alone (A) and ADSCs seeded in ADM (C, D). Red lines underline the cell shape. Magnification 1000x

ADSCs were efficiently seeded on collagen‐based ADMs. Both classical histology and ESEM imaging demonstrated effective incorporation of ADSCs in Integra® (see Figure 2). These data were also confirmed by images acquired through confocal laser scanning microscope (see Figure 3). Not only ADSCs were perfectly incorporated in the pores of the scaffold but also acquired a homogenous distribution. In particular, ESEM and confocal imaging highlighted cell shape and morphology therefore enabling the visualization of ADSCs perfectly adapted in Integra® porous matrix. Moreover, ADSCs seem capable of active proliferation in ADMs, as underlined by the presence of mitotic figures appreciable after HE stain (Figure 2, Panel E).

**FIGURE 4:**
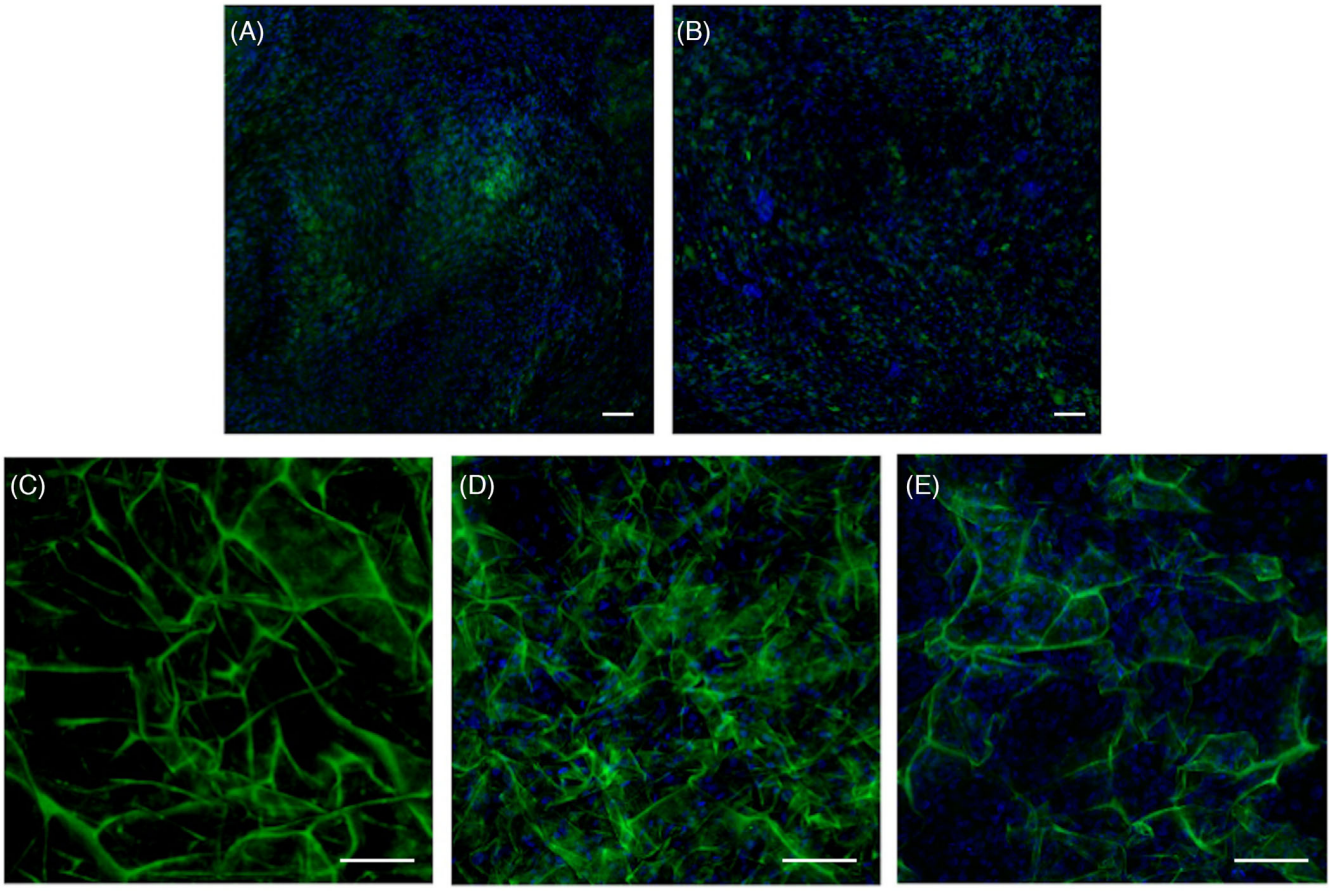
Five representative sections of ADSC‐induced sheet (A), fibroblast‐induced sheet (B), ADM alone(C), ADSC‐seeded ADM (D), and fibroblast‐seeded ADM (E). Magnification 20x. Scale bar: 50 μm

When seeded on Integra®, ADSCs displayed abundant collagen production. ADSC‐seeded ADM was compared not only to fibroblast‐seeded ADM, but also to ADSC‐ and fibroblast‐induced scaffolds and Integra® alone in terms of collagen density (see Figure 4). Collagen production was revealed to be significantly higher when ADSCs were seeded on ADM rather than in all other control conditions.

**FIGURE 5:**
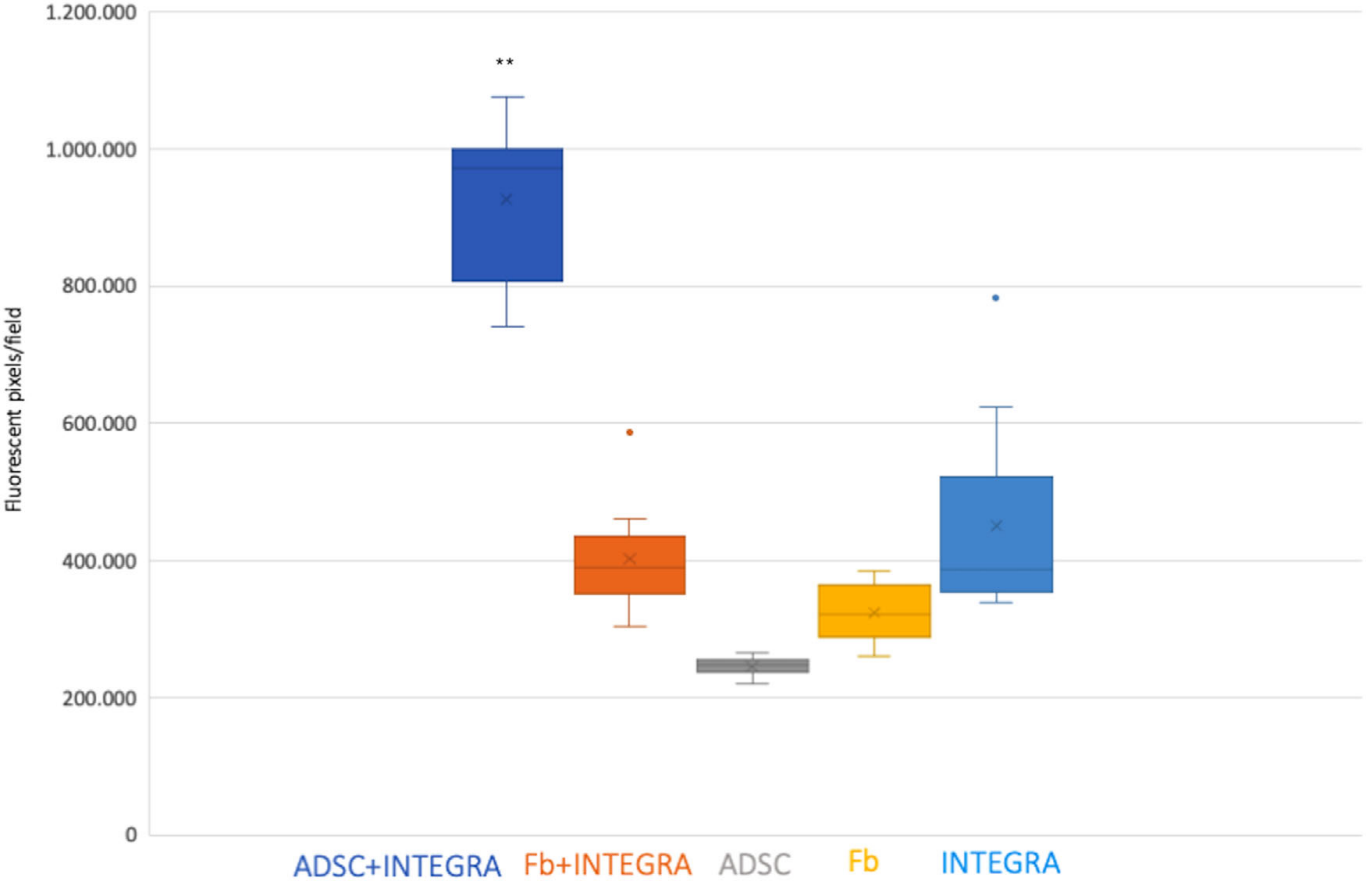
Box plot representing collagen density as mean fluorescence intensity (MFI). MFI is expressed in terms of positive pixels/field, obtained from measurements in three different areas (13 000 μm^2^) of each of three randomly selected sections of every tissue sample. MFI of ADSC‐seeded dermal scaffolds is significantly higher than control conditions (**p < 0.001)

These data were confirmed by semiquantitative analysis of collagen density (represented as MFI, see Figure 5). The presence of Integra® was invariably associated with higher collagen density when compared to cell‐based scaffolds, with ADSCs or fibroblasts alone, in the absence of ADM (*p* < 0.05). However, while Integra® alone and fibroblast‐seeded ADM displayed similar results in terms of MFI, a significant increase in collagen density was achieved exclusively in ADSC‐seeded matrix (*p* < 0.001).

Our results confirm the efficacy of collagen‐based ADMs in providing a guide for cell colonization of the wound bed and tissue repair. Present data also suggest a role for ADMs (Integra® in particular) in promoting and potentiating ADSC regenerative properties.

## 4 DISCUSSION AND CONCLUSIONS

Despite the recent advances in regenerative medicine, complete tissue regeneration still remains a utopic scenario and efforts in tissue engineering are today aimed at limiting scarring as much as possible.^25^ In this setting, scaffolding materials and cell‐based strategies are currently gaining a role of primary importance.

The ultimate goal of scaffold design is to replace natural ECM until host cells can repopulate and synthetize new matrix, and promote neo‐angiogenesis in a physiological manner.^26^ Scaffolds have been shown to control the rate at which cells are introduced into the wound, with some studies suggesting that type‐I collagen sponges are ideal because of their porous structure.^27^, ^28^ Several randomized control trials already demonstrated the efficacy of ADMs in the treatment of full‐thickness skin wounds, venous leg ulcers, and diabetic foot ulcers.^29^, ^30^, ^31^, ^32^ However, most of the available literature focuses on clinical outcomes, like hospitalization and healing time,^33^, ^34^ while studies aimed at evaluating the biological mechanisms underlying ADM action are mostly lacking. At the same time, MSCs have also been described to be crucial for tissue remodeling and homeostasis in the wound healing process and previous data already demonstrated the efficacy of MSC‐based therapies in regenerative medicine, both in the vascular and the cutaneous setting.^16^ However, most of the available literature focuses on the effect of BMSCs, with only few data available on ADSC in the dermatologic setting.

While direct injection of MSCs and the use of biomaterials are both promising approaches to skin defects, they still cannot completely restore skin architecture.^35^ Therefore, combination strategies are today under investigation.^36^

Various materials have been evaluated as possible scaffolds for MSC delivery, including silk fibroin‐chitosan, collagen, chondroitin sulfate, and hyaluronic acid. Nanoparticles and microspheres also have been demonstrated to significantly improve ADSC‐mediated wound healing.^37^, ^38^

A Canadian group assessed the in vitro efficacy of MSCs combined with a commercially available type‐I bovine collagen substrate in experimental models of myocardial disease.^39^ The authors proved that 3D collagen scaffolds improved the regenerative properties of MSCs by enhancing the production of trophic factors and modifying their immunomodulatory and fibrogenic phenotype.

On the other hand, MSCs seem to play a pivotal role in promoting neo‐vascularization of ADMs in full‐thickness wound. A group from Turkey found significant improvements in ADM angiogenesis by combining the effects of negative pressure wound therapy and MSCs.^40^

To overcome some major limitations in using cellular therapies, including long times for in vitro expansion and donor's variability, MSC secretome in the form of conditioned medium (CM) has also been investigated as a possible therapeutic tool.^23^, ^41^ Many authors already proved MSC ability to secrete proepithelizing mediators and recently MSC‐derived factors have also been shown to promote the colonization of collagen‐based 3D scaffolds with human skin cells.^24^ Latter data seem to confirm this role for MSC‐CM in promoting the adherence of keratinocytes and fibroblasts as well as keratinocyte proliferation in collagen‐based scaffolding materials.^42^ Those studies also demonstrated the induction of a prohealing phenotype in fibroblasts cultured on collagen scaffold in the presence of MSC‐CM. Moreover, MSC‐CM contains trophic and proangiogenic factors responsible for keratinocyte and endothelial cell recruitment.

More complex therapeutic strategies aimed at combining MSCs and ADMs with topical drugs are currently under investigation.^43^ The use of timolol in combination with BMSCs and Integra®, for example, already gave excellent results in murine models of diabetic wound healing.^44^

An important aspect of preclinical studies focusing on the combined use of MSCs and ADMs regards imaging techniques for efficacy assessment, since cell integration into scaffolding material is crucial to MSC biological effects. The scarcity of quantitative, nondestructive methods for quality evaluation of engineered tissues poses great limitations to both in vitro and in vivo studies in the regenerative setting.

In 2011, Lutz et al analyzed collagen synthesis and structure using the signals second harmonic generation (SHG) and fluorescence lifetime of collagen autofluorescence by multiphoton laser scanning microscope.^45^ An Indian study focused on laser‐induced autofluorescence as a noninvasive objective tool to measure the endogenous collagen levels in burn wound granulation tissues ex vivo.^46^ A group from California investigated the use of a fiber‐based, multispectral fluorescence lifetime imaging system to nondestructively monitor changes in mechanical properties of collagen hydrogels caused by controlled application of cross‐linking agents.^47^ The authors observed strong and specific correlations between fluorescence lifetime and the storage or Young's moduli of the gels. Lastly, a study group from Beijing also identified different peak values of autofluorescence between scar and normal skin, probably due to different collagen fiber density, providing simple and effective indicators for scar diagnosis and treatment.^48^

Another Chinese group recently used two‐photon excitation fluorescence (TPEF) microscopy and second‐harmonic generation (SHG) microscopy to assess the effects of ADMs re‐cellularized with MSCs in a murine model of wound healing, with impressive results in terms of tridimensional tissue imaging reconstruction.^49^ Moreover, using advanced optical techniques, Wang and coauthors demonstrated that transplanted MSCs differentiate into functional cells and recruit endogenous cells for tissue remodeling in the initial phases after wounding, while only endogenous cells are responsible for the latter stages of cutaneous wound healing.^50^

Also Chu et al studied MSC seeding on ADM in a murine model of diabetic cutaneous wound healing.^51^ Not only the authors demonstrated such treatment to improve angiogenesis and promote reepithelialization, but also significant collagen‐I deposition was detected through the use of multiphoton microscopy and SHG and TPEF imaging.

As mentioned before, we decided to take advantage of collagen autofluorescence. In fact, collagen is one of the main fluorophores of oral mucosa as well as elastin, keratin, and tryptophan.^52^ Other possible fluorophores are porphyrins, advanced‐glycation end products (AGEs), flavins, NADH, FADH, melanin, and lipofuscin.^53^, ^54^, ^55^ From a clinical point of view, an indirect correlation between collagen autofluorescence and cumulative UV exposure was demonstrated. Therefore, a reduction in collagen autofluorescence represents a marker photodamage and denotes deteriorated tissue. Probably, accumulation of AGEs with aging also contributes to tissue stiffness and organ dysfunction by crosslinking ECM proteins like collagen.

Our data give further insights on the in‐vitro interactions between ADSCs and ADMs when used in combination. Not only we demonstrated that ADSCs are capable of efficient integration in collagen‐based ADMs, but we also proved that ADSCs produce collagen and other ECM components even better when seeded in ADMs compared to other control conditions. Moreover, we achieved significant results in collagen deposition assessment and wound healing imaging using collagen natural fluorescence. In fact, sample excitation with a 488‐nm wavelength laser allowed us to obtain 3D reconstructions of the neo‐synthetized tissue and to assess efficacy of MSC integration in collagen‐based ADMs.

In conclusion, present data strongly support the combined use of ADMs and MSCs, with collagen autofluorescence being a potential surrogate marker for their efficacy in wound healing. However, further research in this setting is urgently needed.

## CONFLICT OF INTEREST

The authors report no conflict of interest.
